# A Comprehensive Physiotherapy Approach to Regain Functional Independence in Intermediate Syndrome Secondary to Organophosphate Poisoning: A Case Report

**DOI:** 10.7759/cureus.63929

**Published:** 2024-07-05

**Authors:** Chaitali S Vikhe, Vaishnavi Yadav, Neha A Brahmane

**Affiliations:** 1 Department of Sports Physiotherapy, Ravi Nair Physiotherapy College, Datta Meghe Institute of Higher Education and Research, Wardha, IND; 2 Department of Physiotherapy, Ravi Nair Physiotherapy College, Datta Meghe Institute of Higher Education and Research, Wardha, IND; 3 Department of Pediatric Physiotherapy, Ravi Nair Physiotherapy College, Datta Meghe Institute of Higher Education and Research, Wardha, IND

**Keywords:** physical medicine and rehabilitation, physical therapy, physiotherapy, functional independence, intermediate syndrome, organophosphate poisoning

## Abstract

Organophosphate poisoning (OPP) remains a significant public health issue globally, particularly in middle- and low-income countries. This study aimed to assess the effectiveness of physiotherapy interventions in managing patients with OPP, focusing on reducing the severity of intermediate syndrome symptoms and associated complications such as respiratory muscle weakness and bilateral loculated pleural effusions. A 48-year-old male with a history of alcohol consumption was transferred to the medicine intensive care unit due to poison ingestion. The patient exhibited symptoms of respiratory distress and decreased consciousness, necessitating intubation and mechanical ventilation. Physiotherapy interventions included patient education, secretion mobilization, vital capacity improvement, secondary complication prevention, chest expansion exercises, dyspnea-relieving positions, and mobilization. The patient's progress was monitored using various scales, including the Functional Independence Measure Scale, ICU Mobility Scale, and Chelsea Critical Care Physical Assessment Tool. Significant improvements in functional independence, mobility, and psychological well-being were observed throughout the intervention period. This study highlights the importance of physiotherapy in the comprehensive management of OPP, emphasizing its role in mitigating respiratory complications and improving overall functional outcomes.

## Introduction

Organophosphate poisoning (OPP) is a significant public health concern, particularly in middle- and low-income nations [1]. It can occur through occupational exposure, accidental ingestion, or deliberate self-harm using organophosphate pesticides (OPS) [2]. Despite being cost-effective for agricultural purposes, OPS poses a substantial risk to public health due to their high toxicity. They are notorious for causing severe morbidity and mortality, whether through occupational exposure in agriculture, suicide attempts using pesticides, or their use as nerve agents in warfare [3,4]. Nicotinic stimulation at the sympathetic ganglia and the neuromuscular junction leads to various symptoms, including mydriasis, tachycardia, weakness, hypertension, fasciculations, shallow breathing (diminished respiratory effort), and sweating [5]. Respiratory and cardiovascular failure are common causes of death in OPP, often due to respiratory muscle paralysis and airway obstruction caused by bronchial secretions and bronchospasm induced by OPS [6].

The intermediate syndrome (IMS) significantly contributes to morbidity and mortality associated with OPP due to its common occurrence and the high likelihood of resulting in respiratory failure [7]. It typically manifests 24-96 hours after poisoning. It is characterized by muscle weakness between the acute cholinergic crisis (marked by fasciculations and muscle weakness) and delayed neuropathy caused by the inhibition of neuropathy target esterase [8-10]. In this study, we aim to investigate the effectiveness of physiotherapy interventions in managing patients with OPP, particularly focusing on reducing the severity of IMS symptoms and associated complications such as respiratory muscle weakness and bilateral loculated pleural effusions.

## Case presentation

A 48-year-old male was brought to casualty after consuming poison accompanied by the consumption of alcohol. The patient was an occasional alcoholic. He was found unconscious and had frothing from the mouth, decreased responsiveness, difficulty in breathing, and unstable vitals with a blood pressure of 100/60 mmHg, pulse rate of 102 per minute, and oxygen saturation of 75%. Auscultation revealed bilateral coarse crepitations. The patient was shifted and admitted to the medicine ICU, where he was intubated. All necessary investigations, including an X-ray, were done on admission, and medical management was started with atropine 2 mg, injection of midazolam 2 mg IV, succinylcholine 75 mg IV, and intravenous fluids. On the same day, the patient was referred for physiotherapy.

Patient information

Upon examination, the patient's informed consent was obtained. The patient was unconscious with reduced air entry bilaterally. An endotracheal tube was connected to a mechanical ventilator set to volume control mode with a fraction of inspired oxygen at 100%. The patient's vital signs included a pulse rate of 110 beats per minute, blood pressure of 140/85 mmHg, and a respiratory rate of 27 breaths per minute. A Ryle's tube and Foley catheter were in situ. The respiratory examination revealed decreased bilateral chest movements and bilateral crepitations at axillary and mammary levels. On the second day, the patient was extubated. However, due to worsening conditions, the patient was reintubated four days later, accompanied by the presence of secretions and reduced air entry. After another two days, the patient was extubated again. By the seventh day, the patient was semiconscious and on oxygen support via a face mask. On April 25, 2024, the patient was diagnosed with bilateral pleural effusion over the lower zones. Table 1 provides a timeline of events.

**Table 1:** Timeline of events FIO2: fraction of inspired oxygen; PEEP: positive end-expiratory pressure; IMS: intermediate syndrome

Date	Events
04/17/2024	The patient was brought to casualty after consuming poison and was admitted to the medicine intensive care unit. The patient was on a ventilator, in pressure control mode, with FIO_2_ at 100% and PEEP at 8 cm H_2_O. On the same day, the patient was referred to physiotherapy
04/18/2024	The patient was extubated
04/20/2024	The patient was reintubated, and the ventilator mode was changed to volume control, with FIO_2_ at 70% and PEEP at 6 cm H_2_O
04/22/2024	The patient was diagnosed with IMS
04/23/2024	The patient was extubated, and oxygen support was provided at 6 L/minute
04/24/2024	Oxygen support was reduced to 4 L/minute
04/25/2024	The patient was diagnosed with bilateral pleural effusion
04/27/2024	The patient was transferred to the ward
04/30/2024	The patient was discharged

Investigations

**Figure 1:**
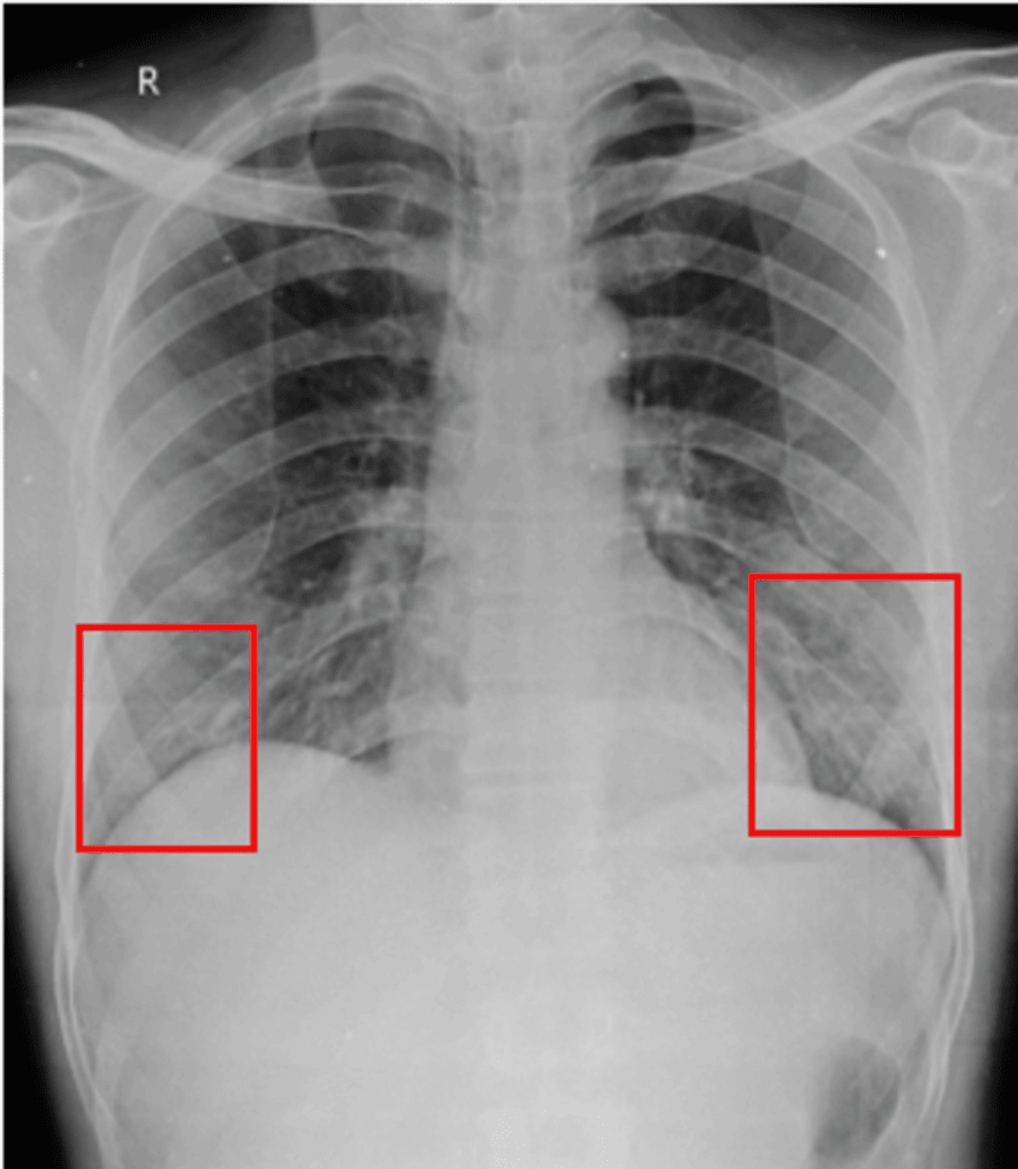
AP view of the chest X-ray taken on day 1 showing bilateral opacity AP: anteroposterior

Thoracic ultrasound showed bilateral pleural effusion, with 260 cc on the left and 80 cc on the right, accompanied by subsegmental atelectasis. Figure 1 shows an anteroposterior (AP) view of the chest X-ray taken on day 1.

**Figure 2:**
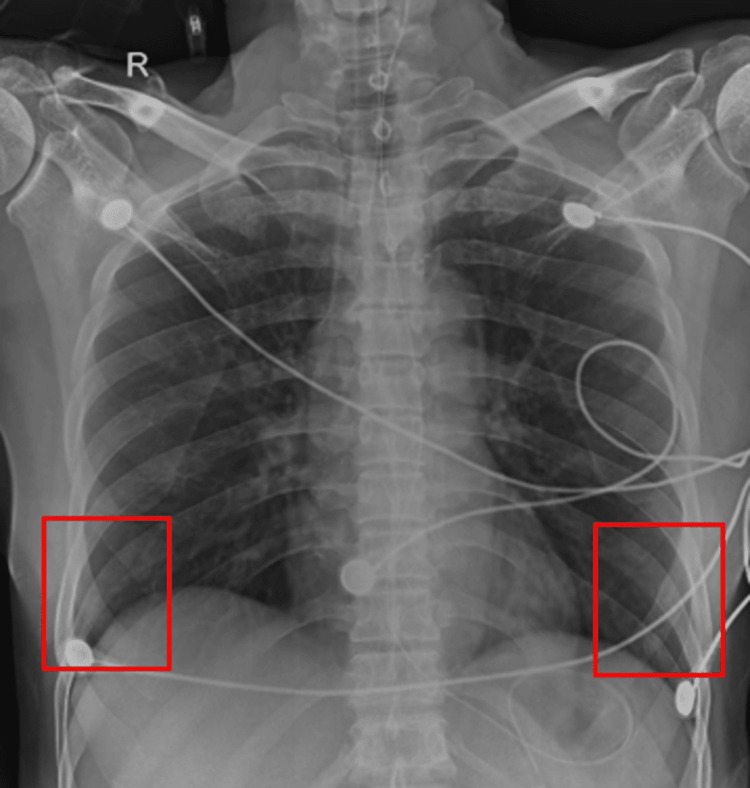
AP view of the chest X-ray taken on day 7 showing translucency AP: anteroposterior

Figure 2 shows an AP view of the chest X-ray taken on day 7.

Physiotherapy intervention

The physiotherapy intervention protocol given for the period of two weeks is shown in Table 2 [11-13].

**Table 2:** Physiotherapy intervention protocol for the period of two weeks N/A: not applicable; PNF: proprioceptive neuromuscular facilitation

Goals	Intervention	Rationale	Frequency	Intensity	Time
Patient education	Educating the patient and family on the importance of exercise and obtaining cooperation and approval from both parties	To ensure understanding and participation in the treatment plan	N/A	N/A	N/A
To mobilize the secretions	Manual chest vibration followed by suctioning when the patient is on a ventilator, positioning three times a day, and an active cycle of breathing technique when the patient is not on a ventilator	To mobilize and clear secretions	Three times a day	Moderate	15 minutes per session
To improve vital capacity	Deep breathing exercises, incentive spirometry three times a day, and chest PNF	To enhance lung function and overall respiratory capacity	Three times a day	Moderate	10 repetitions × two sets
To prevent secondary complications	Upper and lower limb mobility exercises and positioning every two hourly	To prevent complications due to immobility	Every two hours	Low to moderate	10 repetitions × two sets
To improve chest expansion	Thoracic expansion exercises	To enhance chest expansion	Three times a day	Moderate	10 repetitions × two sets
To relieve dyspnea	Dyspnea-relieving positions: high side lying and sit leaning forward	To alleviate shortness of breath	As needed	N/A	N/A
To improve functional mobility	Initiated with sit-to-stand, then ambulation with moderate assistance, and finally independent ambulation	To encourage movement and improve overall mobility	Three times a day	Low to moderate with progression	10 repetitions × one set

**Figure 3:**
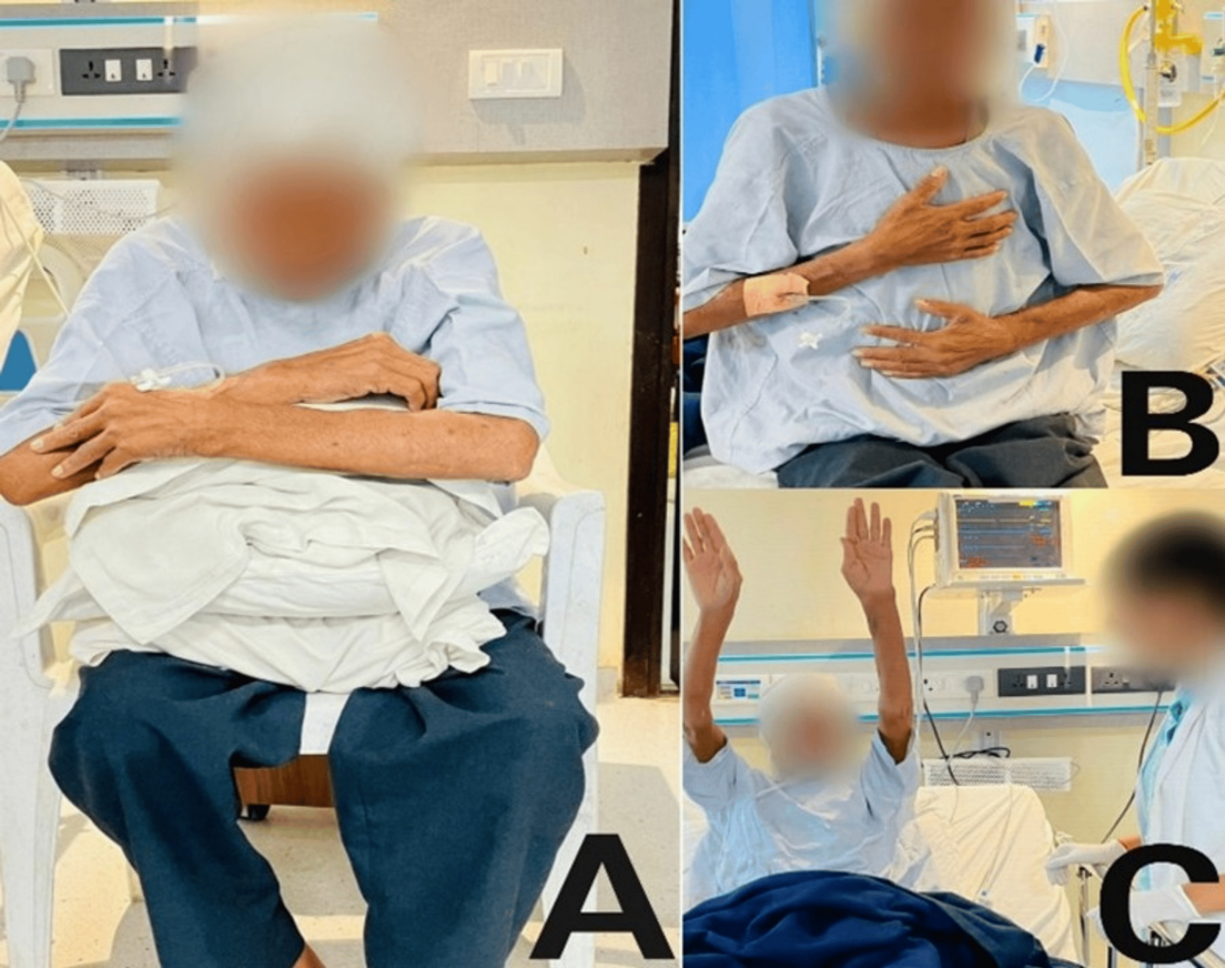
Intervention to enhance lung function and overall respiratory capacity. (A) Forward-leaning position to relieve dyspnea. (B) The patient performing deep breathing exercises. (C) Thoracic expansion exercises to improve chest mobility and expansion

Interventions to enhance lung function and overall respiratory capacity are shown in Figure 3.

**Figure 4:**
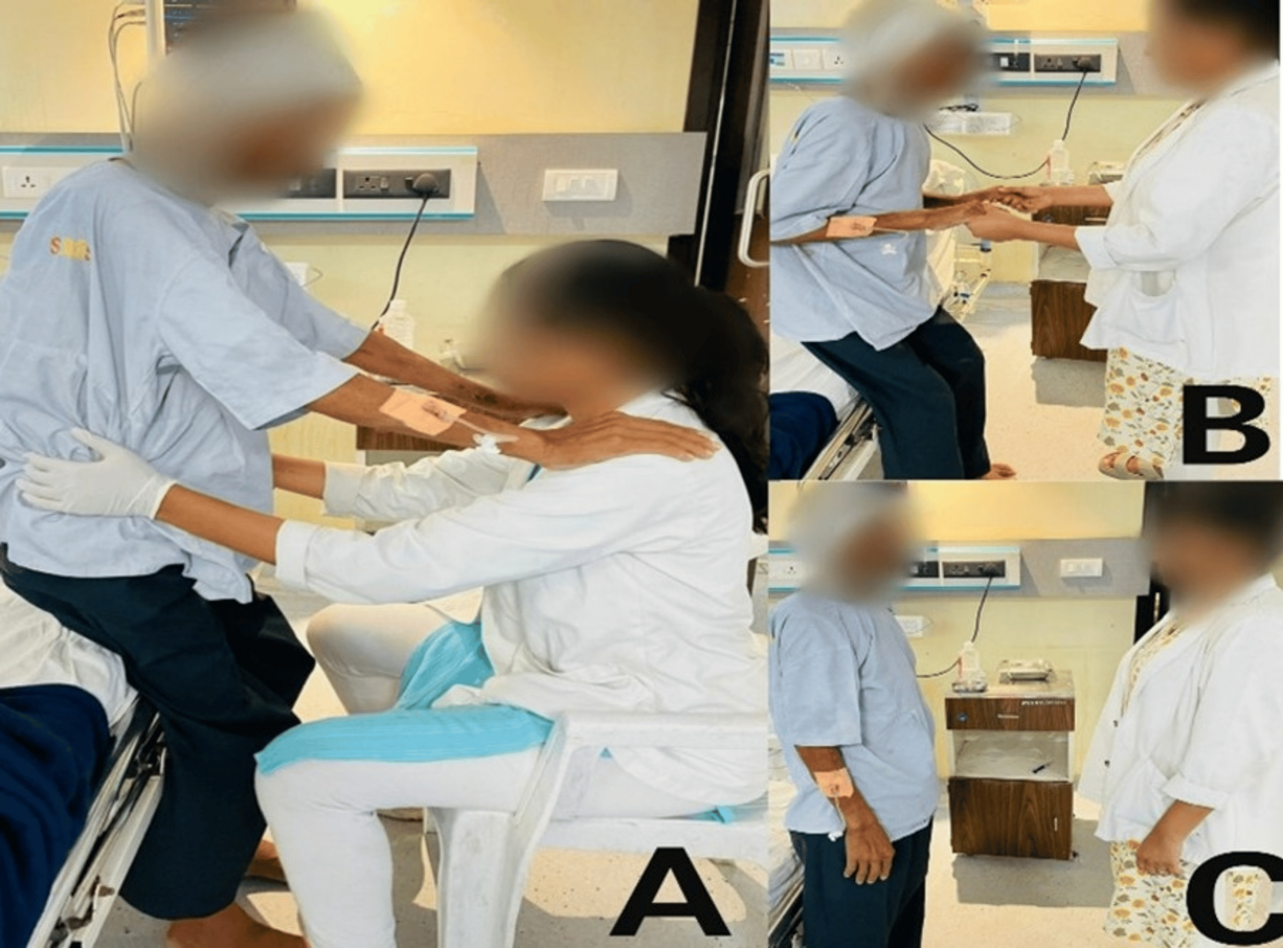
Progression of the patient's mobility. (A) Day 5: sit-to-stand with maximum support. (B) Week 1: sit-to-stand with minimal assistance. (C) Week 2: sit-to-stand without support

The progression of the patient's mobility is shown in Figure 4.

**Figure 5:**
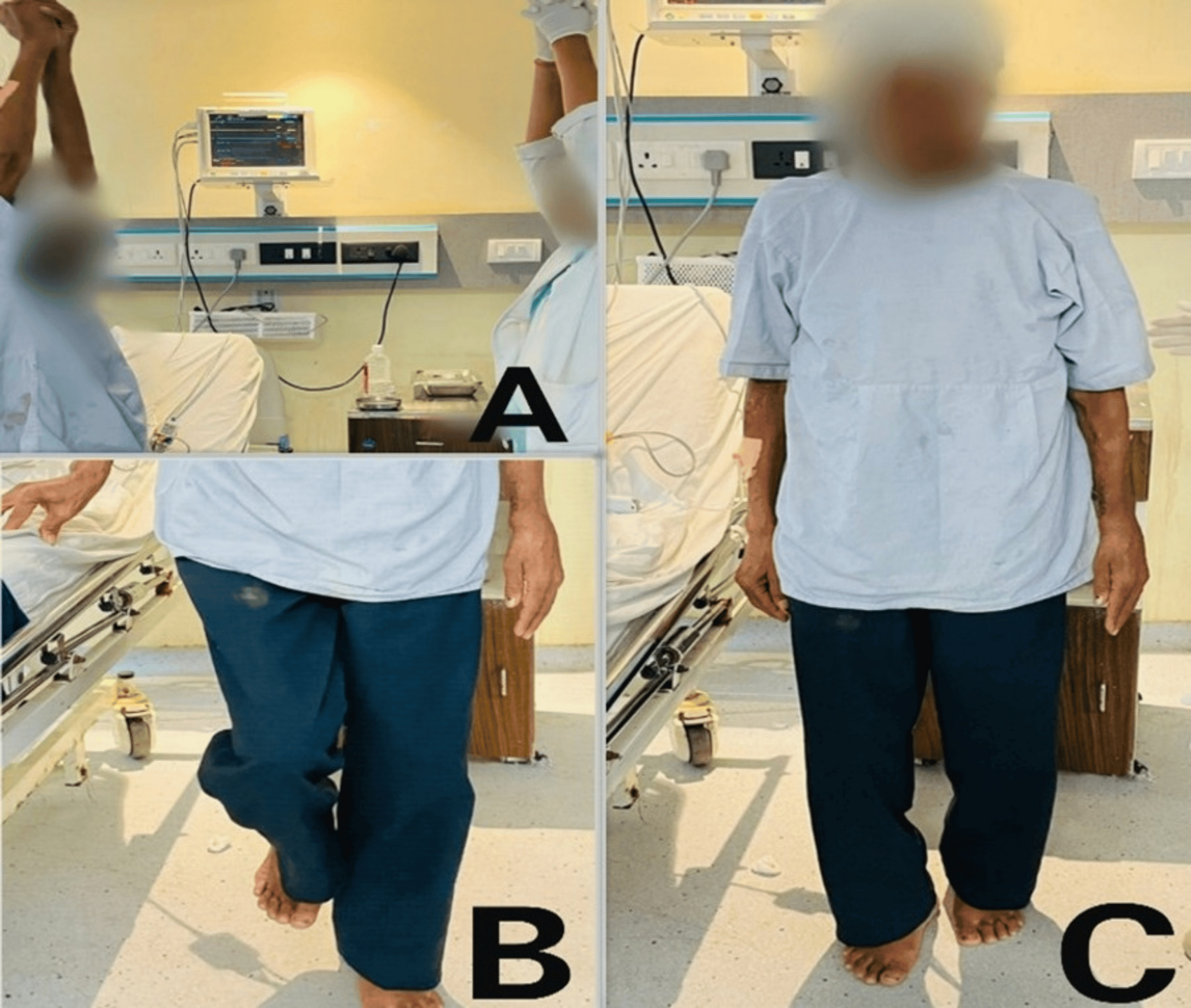
The progression of the patient's physical activities. (A) Upper limb mobility exercises. (B) Spot marching. (C) Week 2: ambulation without any assistance

The progression of the patient's physical activities is shown in Figure 5.

Outcome measures and follow-up

Table 3 presents outcome measures and follow-up assessments across three different scales: the Functional Independence Measure Scale, the ICU Mobility Scale, and the Chelsea Critical Care Physical Assessment Tool. Each scale measures the patient's level of independence or mobility at different time points: day 1, week 1, and week 2. The measures range from maximal assistance to no assistance, from lying in bed to walking without assistance, and from highly dependent to fully independent, as shown in Table 3.

**Table 3:** Progression of patient functional independence and mobility: outcome measures and follow-up

Scales	Day 1	Week 1	Week 2
Functional Independence Measure Scale	Maximal assistance	Minimal assistance	No assistance
ICU Mobility Scale	0 (lying on the bed)	7 (standing without support)	10 (walk without assistance)
Chelsea critical care physical assessment tool [14]	0 (highly dependent and immobile, requiring maximal assistance for all activities)	3 (the patient shows significant improvement, with partial independence)	5 (the patient is fully independent, with restored physical function and mobility)

## Discussion

OPP remains a great public health concern internationally, especially in middle- and low-income countries. The poisoning can be due to different exposures, including occupational, accidental ingestion, or deliberate self-harm through OPS [15,16]. OPS is cost-effective for agriculture, but its high toxicity levels cause tremendous risks correlated with extended morbidity and mortality levels [17,18]. This study aimed to investigate the effectiveness of physiotherapy interventions in managing patients with OPP, specifically in reducing the severity of IMS symptoms and related complications such as respiratory muscle weakness and bilateral loculated pleural effusions. Our findings underscore the critical role of physiotherapy interventions in managing OPP complications. Patient education focused on the importance of exercises to enhance respiratory function and overall health. Techniques for secretion mobilization, such as manual chest vibration and suctioning, maintained clear airways and prevented respiratory complications. The use of incentive spirometry significantly improved vital capacity, indicating enhanced lung function and reduced susceptibility to respiratory issues, which are major mortality factors in OPP. Preventing secondary complications, such as pressure sores and muscle contractures, was achieved through regular positioning and mobility exercises. Thoracic expansion exercises, along with chest proprioceptive neuromuscular facilitation and dyspnea-relieving positions, contributed to better chest expansion and reduced breathing distress, emphasizing the comprehensive role of physiotherapy in managing respiratory complications associated with OPP [19,20].

The patient's progress, monitored using the Functional Independence Measure Scale, ICU Mobility Scale, and Chelsea Critical Care Physical Assessment Tool, showed significant improvements in functional independence, mobility, and psychological well-being. These improvements highlight the holistic benefits of physiotherapy in OPP management, addressing both physical and psychological aspects. The presence of bilateral pleural effusion in this patient indicated a severe and complicated course of OPP, often associated with a poor prognosis. However, the structured and targeted physiotherapy interventions contributed significantly to the patient’s recovery and functional improvements.

## Conclusions

This case report demonstrates the essential role of comprehensive physiotherapy in the effective management of IMS secondary to OPP. The physiotherapy interventions led to significant improvements in the patient's functional independence, mobility, and overall well-being. These results emphasize the necessity of incorporating structured physiotherapy programs into treatment protocols for OPP to mitigate respiratory complications and promote holistic recovery.
